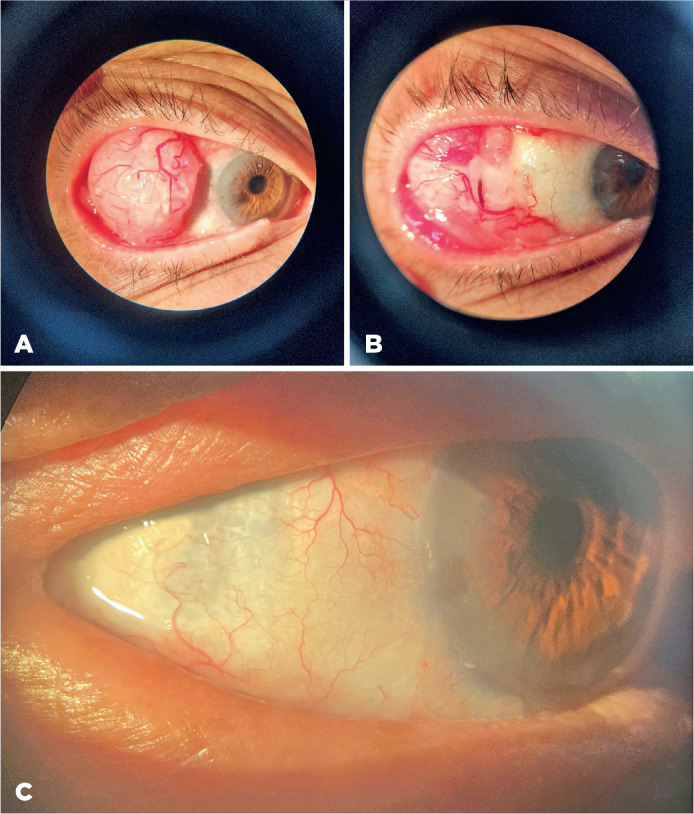# Conjunctival squamous cell carcinoma: risk factors, treatment options, and management

**DOI:** 10.5935/0004-2749.2024-0389

**Published:** 2025-02-11

**Authors:** Andréa Santucci, Nicole Bulgarão Maricondide Almeida, Newton Kara-Junior

**Affiliations:** 1 Ophthalmology Department, Hospital das Clinicas, Faculdade de Medicina, Universidade de São Paulo, São Paulo, SP, Brazil

Conjunctival squamous cell carcinoma (A) is one of the most common ocular tumors. It is typically a unilateral tumor with slow, progressive growth and rare metastasis. Risk factors include ultraviolet (UV) radiation exposure, smoking, human papillomavirus infection, and human immunodeficiency virus infection^(1)^. Treatment options may involve topical chemotherapy agents and immunomodulatory drugs like 5-fluorouracil (5-FU)(B)^(2)^. Intraocular invasion after primary treatment is uncommon and may be prevented by adjuvant brachytherapy following tumor resection (C)^(3)^.

**Figure:**